# Tumor-stroma contact ratio - a novel predictive factor for tumor response to chemoradiotherapy in locally advanced oropharyngeal cancer

**DOI:** 10.1016/j.tranon.2024.102019

**Published:** 2024-06-03

**Authors:** Justus Kaufmann, Maximilian Haist, Ivan-Maximiliano Kur, Stefanie Zimmer, Jan Hagemann, Christoph Matthias, Stephan Grabbe, Heinz Schmidberger, Andreas Weigert, Arnulf Mayer

**Affiliations:** a Department of Radiation Oncology and Radiotherapy, University Medical Center of the Johannes-Gutenberg-University, Mainz 55131, Germany; b Department of Dermatology, University Medical Center of the Johannes-Gutenberg-University, 55131 Mainz, Germany; c Institute of Biochemistry I, Faculty of Medicine, Goethe-University Frankfurt, 60596 Frankfurt, Germany; d Institute of Pathology, University Medical Center of the Johannes-Gutenberg-University, 55131 Mainz, Germany; e Department of Pathology, Stanford University School of Medicine, Stanford, CA 94305, USA; f Department of Microbiology & Immunology, Stanford University School of Medicine, Stanford, CA 94305, USA; g Department of Otorhinolaryngology, University Medical Center of the Johannes-Gutenberg-University, Mainz 55131, Germany; h Division of Radiation Oncology, Peter MacCallum Cancer Centre, Melbourne, Australia

**Keywords:** Head-and-neck cancer, Oropharyngeal squamous cell carcinoma, Pattern of invasion, Tumor microenvironment, Spatial tumor biology, Tumor stroma interaction, multiplex immunohistochemistry, Chemoradiotherapy

## Abstract

The growth pattern of oropharyngeal squamous cell carcinomas (OPSCC) varies from compact tumor cell aggregates to diffusely infiltrating tumor cell-clusters. The influence of the growth pattern on local tumor control and survival has been studied mainly for surgically treated oral cavity carcinomas on a visual basis. In this study, we used multiplex immunofluorescence staining (mIF) to examine the antigens pan-cytokeratin, p16INK4a, Ki67, CD271, PD-L1, and CD8 in pretherapeutic biopsies from 86 OPSCC. We introduce Tumor-stroma contact ratio (TSC), a novel parameter, to quantify the relationship between tumor cells in contact with the stromal surface and the total number of epithelial tumor cells. mIF tumor cores were analyzed at the single-cell level, and tumor-stromal contact area was quantified using the R package "Spatstat". TSC was correlated with the visually assessed invasion pattern by two independent investigators. Furthermore, TSC was analyzed in relation to clinical parameters and patient survival data to evaluate its potential prognostic significance.

Higher TSC correlated with poor response to (chemo-)radiotherapy (*r* = 0.3, *p* < 0.01), and shorter overall (OS) and progression-free (PFS) survival (median OS: 13 vs 136 months, *p* < 0.0001; median PFS: 5 vs 85 months, *p* < 0.0001). Visual categorization of growth pattern according to established criteria of tumor aggressiveness showed interobserver variability increasing with more nuanced categories (2 categories: *k* = 0.7, 95 %-CI: 0.55 - 0.85; 4 categories *k* = 0.48, 95 %-CI: 0.35 - 0.61).

In conclusion, TSC is an objective and reproducible computer-based parameter to quantify tumor-stroma contact area. We demonstrate its relevance for the response of oropharyngeal carcinomas to primary (chemo-)radiotherapy.

## Highlights

• Tumor-stroma contact surface (TSC) can be used as a surrogate parameter for tumor stroma interaction in multiplex immunofluorescence images.
• TSC correlates with response to chemoradiotherapy in oropharyngeal squamous cell carcinoma.
• Prognosis, especially of p16-negative oropharyngeal squamous cell carcinomas, is correlated with size of the TSC.
• Decreased infiltration of CD8+ cytotoxic T-Lymphocytes correlates with larger TSC.

## Introduction

Head and neck squamous cell carcinomas (HNSCC) represent the sixth most common cancer worldwide and are characterized by a high rate of local recurrence and metastatic dissemination [1]. Several parameters that constitute a “high risk”-situation for local recurrence in the postoperative setting such as depth of infiltration, resection margins, worst pattern of invasion (WPOI) and extranodal tumor spread from lymph node metastases have been identified [2,3]. There is, however, a lack of prognostic markers to aid in the decision-making process regarding the primary treatment modality for locally advanced OPSCC. The identification of new parameters predictive for response to either radical surgery or definitive chemoradiotherapy is therefore crucial to make the optimal choice for individual patients.

While data on the pattern of invasion in OPSCC is sparse, extensive evidence from squamous cell carcinomas of the oral cavity (OSCC) demonstrates that invasive growth patterns at the tumor-host border are associated with poorer outcomes, higher metastatic risk, and reduced survival [[4], [5], [6], [7], [8], [9]]. Consequently, risk stratification scores incorporating invasion patterns have been proposed to guide adjuvant radiotherapy decisions in OSCC [3,4]. Despite comparative studies demonstrating the superior prognostic value of grading systems that integrate invasion patterns, these factors have not yet been adopted by the WHO grading system, which still relies primarily on differentiation-based parameters proposed by Broders in 1920 [[10], [11], [12], [13], [14], [15], [16], [17]].

The tumor-stroma interface has emerged as a critical determinant of cancer progression and treatment response across various solid tumors and there is increasing support for the hypothesis that the pattern of invasion at the tumor-host border is, in part, driven by tumor stroma interaction within the tumor [18,19]. We hypothesized that a larger tumor-stroma contact surface would result in higher chances of interaction between tumor cells and stromal cells, which might be associated with more aggressive phenotypes. To test this hypothesis, we developed a novel single-cell based classification approach and applied spatial statistics to quantify the tumor-stroma contact surface in OPSCC patients. We introduce the "tumor stroma contact ratio" (TSC), defined as the proportion of epithelial tumor cells at the tumor-stroma border relative to all epithelial neoplastic cells, as an objective surrogate parameter for the extent of tumor-stroma interaction. To our knowledge, this is the first study to objectively quantify the tumor-stroma contact surface in OPSCC using computational biology methods and to evaluate its prognostic and predictive value in the setting of primary chemoradiotherapy.

## Patients and methods

### Patient cohort

Details on clinical and pathological parameters at initial diagnosis, subsequent treatments and survival data were collected from the patients’ medical records and are described elsewhere [20]. In brief, 476 patients treated for primary HNSCC between 2005 and 2019 at the University Medical Center Mainz, Germany, were screened. 86 patients with survival follow-up data until April 2022 were retrospectively identified according to the following selection criteria: diagnosis of squamous cell carcinoma of the oropharynx (all subsites), absence of secondary HNSCC tumors diagnosed within the observation period, pre-treatment tumor tissue available for the generation of a TMA, >30 % of uncompromised tumor tissue on TMA for each patient, treatment with primary radiotherapy or chemoradiotherapy (RT/RCTx), complete follow-up documentation of treatment outcomes..

### Generation of tissue microarray

Of the entire cohort of 475 patients, all 86 patients who met the selection criteria and for whom one or more paraffin tissue blocks were available were included in the present study. Formalin-fixed paraffin-embedded tissue blocks of these 86 OPSCC patients were retrieved from the archives of the Institute of Pathology. The area of malignancy was marked by a pathologist (S.Z.). The tissue microarrays (TMAs) were constructed from 1.2 mm diameter cores that were punched from two representative regions of the tumor tissue blocks according to standard procedures.

### Antibody screening, validation and titration for multiplex immunofluorescence staining and multiplex immunofluorescence staining

The exact procedures for screening, validation and titration of antibodies has been described previously [21,22]. Seven-color multiplex fluorescence staining of the TMA for the antigens pan-Cytokeratin (panCK) AE1/AE3, p16INK4A (p16), CD271, PD-L1, Ki67 and CD8 was performed using the Opal Polaris 7-Color Manual IHC Detection Kit according to the manufacturer's instructions. In brief, after cutting 3 µm thick sections with high precision microtomes, specimens were incubated at 60 °C for one hour and deparaffinized in a descending alcohol series. Pretreatment for multiplex immunofluorescence (mIF) was carried out using antigen-demasking buffers specific for the antigen chosen in each staining round. The subsequent staining process was performed six times in a serial fashion. Sections were incubated with the antibody diluent for 10 min at room temperature, followed by incubation with the primary antibody either for 60 min at 29 °C or overnight at 4 °C. After applying Opal polymer horseradish peroxidase-conjugated secondary antibody and Opal fluorophore solution each for 10 min, antibodies were removed by microwave treatment (heat-induced epitope retrieval; HIER) before a further round of staining. Finally, the nuclei were counterstained with DAPI and after rinsing with PBS, samples were covered with a coverslip using a fluorescence mounting medium. The antibodies, their dilutions, the according retrieval buffers as well as the sequence of usage are described in supplementary Table 1 and 2. The seven-color Opal slides were visualized using the Vectra Polaris Automated quantitative Pathology Imaging System and spectral unmixing was applied to distinguish between fluorescence signals. Detailed images and analysis of fluorescence markers not discussed in this manuscript have been published elsewhere [20].

### Definition of tumor growth pattern phenotypes

**Fig. 1:**
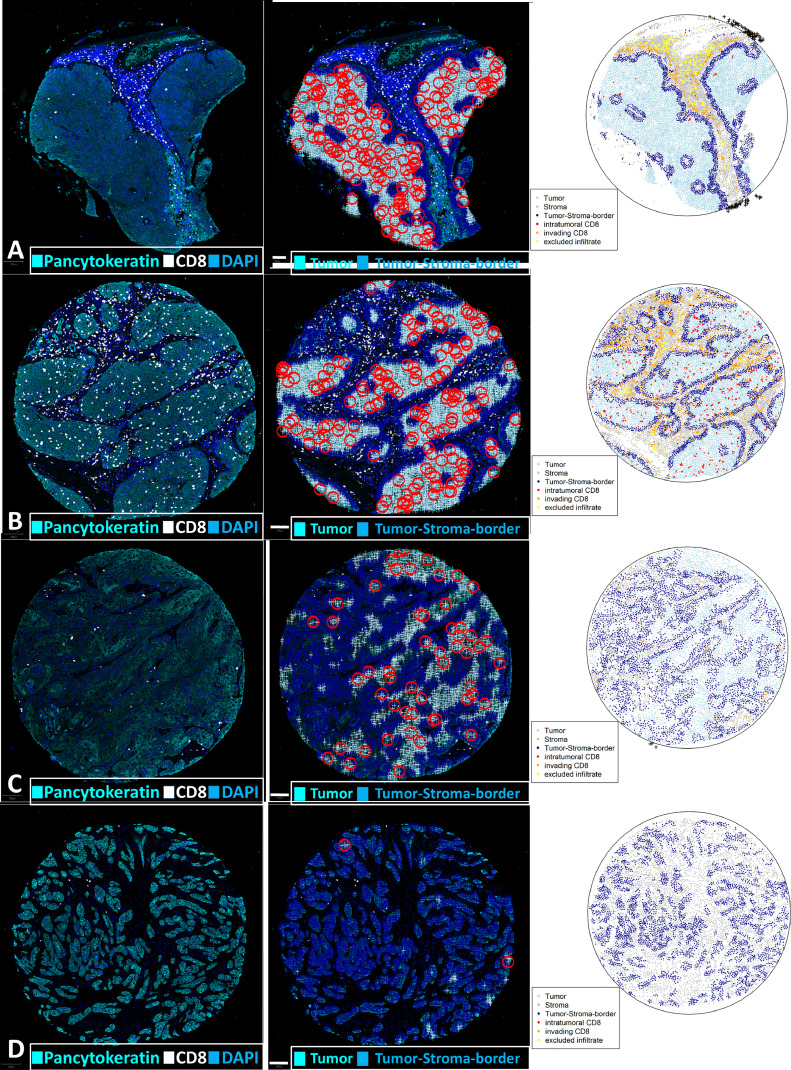
More aggressive growth patterns are correlated with a higher TSC. Left column: Original fluorescence image. Channels shown: Cyan -Pan-CK; White: CTL; Dark blue: DAPI. Middle column: Merge of point pattern data and original immunofluorescence. Light blue marks show tumor cells that are not considered part of the tumor stroma contact surface. Dark blue marks show tumor cells at the tumor stroma contact surface. Circles in red show a 35 µm diameter around random exemplary tumor cells not defined as part of the tumor stroma contact surface. Circles do not show overlap with stromal cells. Right column: Corresponding point patterns. Both degree of invasiveness (see section 2.4), as assessed visually, as well as TSC increase from top to bottom, panels A to D. Scale bar: 100 µm.

Tumor growth patterns were defined in 4 categories ranging from “very bulky” to “very invasive” phenotypes (Fig. 1A-D). Due to low interobserver agreement we decided to simplify the categorization into “bulky” or “invasive”. “Bulky” (Fig. 1A+B) was used if the tumor cells were observed within large tumor cell aggregates that were surrounded, but not infiltrated by stromal tissue, while “invasive” referred to a more diffusely infiltrating phenotype (Fig. 1C+D). While a scoring system for the pattern of invasion at the tumor/normal tissue front exists, there is currently no such score for the growth pattern within the tumor itself [3,18]. Therefore, the definition of phenotypes was based on scoring systems for the “worst pattern of invasion” and the compactness of tumor cell bulks. Patterns of invasion were defined independently by two observers blinded to the patients’ survival data and p16 status.

### Quantitative analyses

Single-cell-based analyses were carried out for all TMA cores with a preserved tumor tissue >30 % using the DAPI channel (blue) for the segmentation of cell nuclei in the open-source whole slide image analysis software QuPath, as described previously [https://qupath.github.io/;[23]]. Further subclassification of cells in distinct phenotypes was based on the relative expression of the defining markers using a thresholding-based classification approach. In brief, cells were classified as „tumor“ or „stroma“ based on the marker panCK, as well as a number of morphological parameters, e.g. cell shape and nuclear-cytoplasmic ratio, using a random-forest machine learning algorithm. Further subclassification was performed either by intensity thresholding for markers with a nuclear expression (Ki67 and p16) or through an object-based training algorithm for membranous staining patterns (CD8, CD271 and PD-L1). This was conducted by two independent observers, blinded to the patients’ survival data, who performed the quantification analysis and classified cell types.

### Spatial analysis

Spatial subclassification of the tumor cell compartment was carried out using the R-package “Spatstat” [https://spatstat.org/;[24]]. The procedure for import and subclassification of cells in R has been described in detail previously [25]. In other works, the invasive tumor front - where tumor stroma interaction is expected to happen - is described as the first three to six cell layers of a tumor cell aggregate [26,27]. Since we worked with single cell data, we defined the tumor stroma contact surface as tumor cells within a distance of 35 µm of any stromal cell. Resulting plots of the marked point patterns were assessed visually and showed a biologically plausible tumor stroma contact surface (Fig. 1A-1D, Suppl. Fig. 1). Cytotoxic T lymphocytes (CTL) were classified similarly into intratumoral, stromal and “marginal infiltrate”. The “marginal infiltrate” was defined as CTL with a distance of less than 35 µm to both tumor and stroma cells. The tumor stroma contact ratio (TSC) was defined as the number of cells at the tumor stroma contact surface divided by the total number of epithelial tumor cells within the observed biopsy.

### Statistical analysis

As a primary time-to-event endpoint, this retrospective cohort study used overall survival (OS), which was estimated using the Kaplan–Meier product-limit method and log-rank statistics in R. Progression-free survival (PFS) was defined as the time interval from start of radiation therapy to physician-reported date of progression, relapse, death date or start date of a new treatment due to progression of disease (whichever event occurred first). PFS was used as a secondary time-to-event endpoint of this study and was estimated in a similar fashion to OS. The association between tumor response (complete response + partial response vs stable disease + progressive disease) and the quantitative IF data was analyzed using the student's *t*-test and Spearman's Rho. In all cases, two-tailed p-values were calculated and considered significant with a value of *p* < 0.05. R (Version 4.0.3; [28]) and RStudio (Version 1.3.1093; [29]) were used for all analyses. The R-package “spatstat” was used to perform cell-cell-distance analyses and define spatial sub-compartments.

## Results

### High tumor stroma contact ratio correlates with invasive growth pattern and is effective in predicting poor response to chemoradiotherapy

Among the 86 OPSCC patients, we observed a strong heterogeneity in terms of tissue architecture, immune cell infiltration and tumor marker expression. In terms of tissue architecture, we could however identify two main phenotypes, defined by the tumor stroma contact ratio (TSC). As mentioned in section 2.5, tumor cells with a distance of less than or equal to 35 µm to the nearest stroma cell were classified as being part of the tumor stroma contact surface. While in some cases of the TSC was as little as 16 % of the observed tumor cells (“bulky tumor phenotype”), in other cases TSC was over 99 % of tumor cells (“diffusely-infiltrating phenotype”) with a median TSC of 62.2 %.

**Fig. 2:**
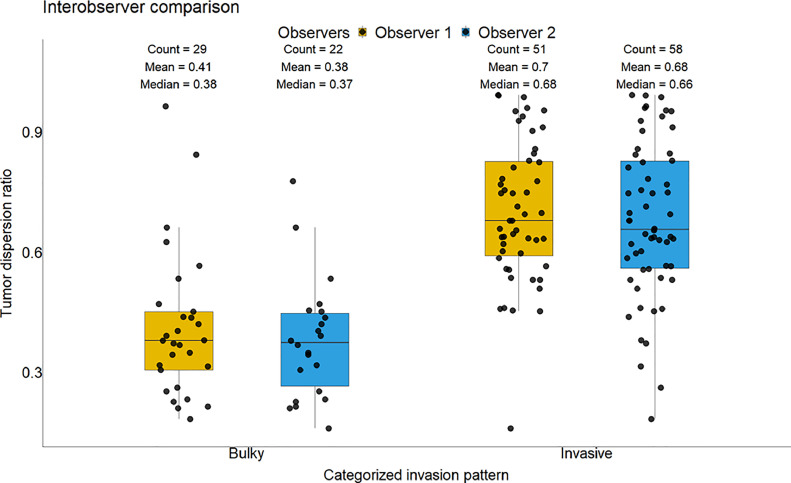
Boxplot demonstrating the correlation between TSC and different growth patterns as defined by different observers (yellow: observer 1; blue: observer 2).

Regarding observer grading of tumor phenotypes, Cohen's kappa test showed substantial inter-observer agreement (0.7, 95 %-CI: 0.55 - 0.85) for invasion grading into the two categories “bulky” and “invasive”, but only moderate to low interobserver agreement for more nuanced invasion grading (*k* = 0.48, 95 %-CI: 0.35 - 0.61). The visual analysis of the growth pattern, performed independently by two observers (JK and MH), showed a strong correlation with the computerized, objective TSC. This correlation was evident in the individual observer analyses (Observer 1: *r* = 0.64, *p* < 0.001; Observer 2: *r* = 0.6, *p* < 0.001) (Fig. 2) and in a pooled analysis combining both observers' assessments (*r* = 0.69, *p* < 0.001).

**Fig. 3:**
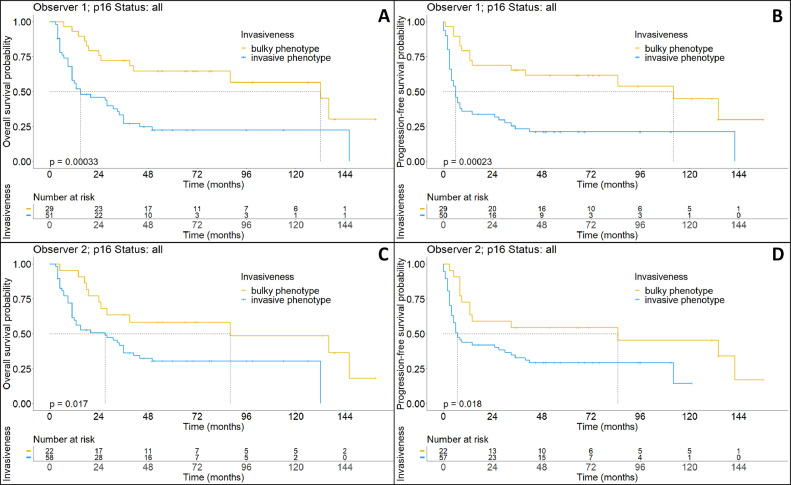
Upper row: Kaplan-Maier plots showing overall (A) and progression-free (B) survival for different phenotypes as defined by observer 1. Lower row: Kaplan-Maier plots showing overall (C) and progression-free (D) survival for different phenotypes as defined by observer 2. While a significant trend can be seen in all cases, there is significantly increased impact by the analysis of observer 1.

Significant differences in survival could be seen for both observers (Observer 1: median OS: 15 vs 132 months, *p* < 0.001; median PFS: 6 vs 112 months, *p* < 0.001; (Observer 2: median OS: 27 vs 88 months, *p* < 0.05; median PFS: 85 vs 7 months, *p* < 0.05), results from observer 1 however showed more impact of growth type on both OS and PFS, while the invasion grading of observer 2 showed a significant, but smaller, impact on patient outcome (Fig. 3).

**Fig. 4:**
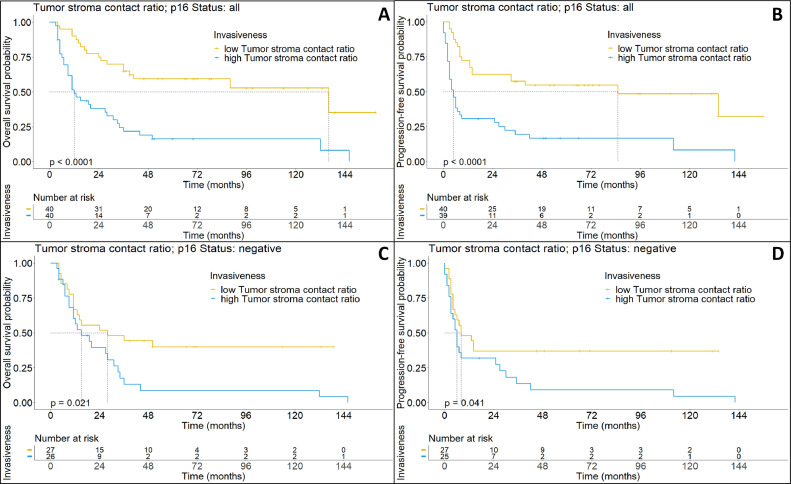
(A) Kaplan-Meier-plot describing the overall survival of all patients dichotomized by median TSC. (B) Kaplan-Meier-plot describing the progression-free survival of all patients dichotomized by median TSC. (C) Kaplan-Meier-plot describing the overall survival of all patients with p16-negative OPSCC dichotomized by median TSC. (D) Kaplan-Meier-plot describing the progression-free survival of all patients with p16-negative OPSCC dichotomized by median TSC.

While there was a clear correlation between “bulky” phenotypes and improved OS, the objective measure of TSC showed an improved discriminatory power in comparison. We observed that higher TSC correlated strongly with poorer outcomes and was highly significant with regard to disease progression when dichotomized at the median (median OS: 13 months vs 136, *p* < 0.0001 and median PFS: 5 months vs 85 months, *p* < 0.0001) (Fig. 4A and 4B).

### p16-positive OPSCC have a proportionally smaller tumor stroma contact surface compared with p16-negative OPSCC

We observed that the TSC was significantly higher in p16-negative OPSCC as compared to their p16-positive counterparts. While, there was a wide range of growth patterns in both p16 positive and p16-negative tumors (minimum in p16-positive OPSCC 18 %, max 98 %; minimum in p16-negative OPSCC 16 %, max 99 %), our results revealed that in p16-positive OPSCC the median TSC was 45.44 %, while in p16-negative OPSCC the median TSC was 65.42 % (*p* = 0.011). Notably, TSC showed the strongest prognostic effect in p16-negative patients (median OS: 15 months vs 28 months, *p* < 0.03 and median PFS: 6 months vs 8 months, *p* < 0.05) (Fig. 4C and 4D), while for p16-positive OPSCC tumors we did not detect a significant survival benefit for patients with low TSC, although this was probably due to low sample size (data not shown).

### Best overall response correlates with tumor stroma contact surface

As a higher TSC suggests a more invasive growth pattern, we hypothesized that microscopic phenotypes with higher TSC might be associated with a higher degree of local treatment failure. We could show a significant difference in TSC between patients with (complete or partial remission) and without (stable or progressive disease) treatment response (Mean 0.55 ± 0.06 vs 0.67 ± 0.08; *p* < 0.02). Furthermore, there was correlation between TSC and best overall response (*r* = 0.3, *p* < 0.01)

### A larger tumor stroma contact surface correlates with decreased intratumoral infiltration of CD8+ CTL

The tumor stroma interface is considered a central compartment for orchestrating anti-tumor immune response. Therefore, we have additionally investigated CTL infiltration within this compartment using spatial analysis.

**Fig. 5:**
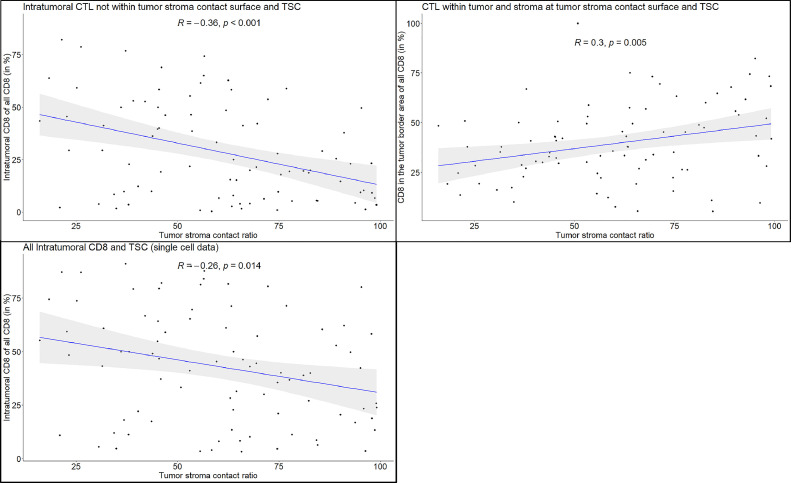
Top left: Correlation between TSC and the percentage of tumor-infiltrating CTL out of all CTL. Increased TSC correlates with reduced number of tumor-infiltrating CTL. Top right: Correlation between TSC and the percentage of CTL that are within 35 µm of the tumor stroma contact surface. With an increasing TSC the size of the “marginal infiltrate” grows. Bottom: Correlation between all tumoral CTL and TSC without further spatial subclassification within the epithelial tumor cell compartment.

We observed, that a larger TSC was correlated with a locational shift of CTL from intratumoral infiltration (*r* = −0.3; *p* < 0.01) towards a “marginal infiltrate”, i.e., CTL within either tumor or stroma located within 35 µm of the tumor stroma contact surface (*r* = 0.3; *p* < 0.01). One might argue that this may be since there is a shift in the ratios of tumor cells and tumor cells in contact with stroma with increasing TSC. However, when CTL were categorized solely by their location to either intratumoral or stromal positions, we also observed a significant negative correlation between CTL within the epithelial tumor cell compartment (including the intratumoral cells of the “marginal infiltrate”) and TSC (*r* = −0.26; *p* < 0.05) (Fig. 5). This suggests that the increase in CTL at the tumor stroma interface is mainly caused by CTL found in the stroma, indicating an "excluded infiltrate".

### Response to chemoradiotherapy and TSC are not affected by the degree of CD271, PD-L1 nor Ki67 within the tumor stroma contact surface

Although our results revealed a significant inverse correlation between the TSC and CTL infiltration and there is an association of a tumor's TSC and its response to chemoradiotherapy, we were unable to detect other factors mediating this effect. Factors that are considered reasons for a more invasive tumor are its proliferation rate, which is also proposed to be influenced by the amount of tumor stem cells. One potential stem cell marker in OPSCC is CD271 [30,31].

There was no correlation between TSC and the relative expression of CD271, Ki67- or PD-L1 within neither the tumor stroma border nor the whole epithelial tumor cell compartment. In addition, expression of PD-L1 (Median 29.1 % ± 28.8% vs 30.0 % ± 26.6 %, *p* = 0.71), Ki67 (Median 32.7 % ± 17.1% vs 30.9 % ± 17.1 %, *p* = 0.75) or CD271 (Median 54.3 % ± 31.1% vs 37.4 % ± 31.3 %, *p* = 0.07) in the tumor stroma contact area was similar in tumors with and without a response to treatment.

## Discussion

Extensive in vivo data from human carcinomas demonstrates the critical role the activated tumor stroma plays in tumor growth and treatment response. In particular, the pattern of tumor invasion, driven by interactions between malignant epithelial cells and activated fibroblasts, creates particular tumor microenvironments (TME) and reflects the invasive potential of squamous cell carcinomas (SCC) of the head and neck region [[4], [5], [6], [7], [8],[11], [12], [13], [14]]. Hence, it would be desirable to have an objective, reproducible and quantitative measure representing the shape of the tumor-stroma interface to enable correlation with other pathological characteristics of the tumor (e.g., biomarker expression or immune cell composition) and clinical outcome data.

Our study, for the first time, provides a novel parameter to quantify the tumor-stroma interface in an objective manner, which we refer to as the tumor-stroma contact ratio (TSC). Higher TSC, indicative of more infiltrative growth, correlated with poorer outcomes, weaker treatment response, and decreased immune infiltration into the epithelial tumor cell compartment.

We first performed a conventional visual assessment of the specimen. Even this coarse, traditional analysis demonstrated a poorer prognosis for more invasive growth patterns in OPSCC. However, investigators disagreed in some cases, highlighting the subjectivity of visual assessment alone. By providing an unbiased and efficient method for risk stratification, TSC resolves interobserver variability and allows more objective treatment discussions.

As expected, HPV-associated OPSCCs, known for their basaloid morphology and compact growth, had lower TSC values compared to HPV-negative tumors. HPV positive tumours with a high TSC had poorer progression free survival, but this was not statistically significant. Possible explanations include the small sample size and intrinsic treatment sensitivity of HPV-associated disease overriding effects of invasion patterns. Conversely, TSC significantly correlated with response and survival in HPV-negative patients, underscoring its clinical relevance in this distinct disease entity.

Although it would be preferable to perform additional in vivo experiments elucidating the underlying molecular mechanisms of different growth patterns, the creation of multiple animal models required for such an endeavor is something beyond the scope of our current study. Several possible reasons for the increased aggressiveness and therapy resistance of a tumor exhibiting a worse pattern of invasion come to mind. First, a more invasive phenotype allows for easier tumor spread into the surrounding tissue and is associated with increased risk of metastasis [32]. This tumor spread is made possible by a remodeling of the extracellular matrix by cancer-associated fibroblasts (CAF) through the activation of Rho- and ROCK-dependent actomyosin interactions [33,34]. There is also evidence suggesting that DNA damage repair pathways are associated with certain growth patterns [35].

Second, more intense communication with the surrounding stroma could allow cancer cells to more easily manipulate the microenvironment and to suppress an antitumor immune response [[36], [37], [38]]. Microenvironment changes caused by tumor stroma interaction can be manifold. Invasive tumors may exhibit regions of increased hypoxia (low oxygen levels). The relationship between invasiveness and hypoxia is complex and varies with tumor characteristics and microenvironment [39,40]. Some studies link invasive tumors to increased hypoxia due to abnormal vasculature and higher metabolic demand, while others suggest better oxygen access due to proximity to blood vessels [[41], [42], [43], [44]]. Hypoxia is known to enhance radioresistance by impairing DNA-damaging free radical generation during radiotherapy and activating survival and DNA repair pathways [45,46]. Furthermore, secretion of growth factors such as TGF-β or HGF by CAFs drives epithelial-to-mesenchymal-transition of tumor cells, while stromal-derived factor 1 increases tumor growth and neoangiogenesis [19,[47], [48], [49]].

The impact of heightened TGF-β secretion has been shown in several experiments with rodent gland-free mammary fat pad assays, where tumor induction was shown to be dependent on either activated fibroblasts or carcinogenic treatment of the stroma before implantation of non-tumorigenic epithelial cells [[50], [51], [52], [53]]. In addition, extensive mouse studies could show that these tumor stroma interactions can play a key role in the sensitivity of cancers to specific cancer-targeted therapy [54]

Third, closer proximity to blood vessels and stromal cells would facilitate both the transfer of microvesicles and metabolic coupling, thereby creating enhanced conditions for tumor cell growth [55,56].

The role of the phenotype and spatial distribution of tumor-infiltrating lymphocytes for response to treatment and prognosis is currently in the focus of immunological research in HNSCC [[57], [58], [59], [60]]. Mechanisms mediating these spatial behaviors are of significant interest for future therapy strategies. Here, we could show that the tumor stroma interface might be a critical compartment for orchestrating anti-tumor immune responses. TSC significantly correlated with decreased immune infiltration into the tumor epithelial compartment and also with an increased "excluded infiltrate" at the tumor-stroma interface [61]. Similar findings have been described by other authors [62].

CD271 has been shown to be a stem cell marker in other HNSCC sites with a strong expression within the invasive front of a tumor [63,64]. However, so far we were unable to find evidence demonstrating the same role in OPSCC [20]. Therefore, it is not surprising that we did not see a significant correlation between TSC and CD271.

To our knowledge, this is the largest cohort consisting of OPSCC patients evaluated for patterns of invasion [5]. In addition, this is a homogeneous patient cohort consisting entirely of locally advanced (i.e. T3/4, *N*+, M0) OPSCC treated with primary definitive (chemo-)radiotherapy according to relatively uniform treatment protocols. Although the retrospective nature of this study is a limitation, retrospective data are on a high level of quality due to detailed documentation in electronic patient files in combination with scheduled follow-up meetings according to German radiation protection laws. Finally, our cohort has a long median follow-up time that allows for the detection of insidious late distant recurrences which can be observed in some HPV-associated cases.

By investigating the relationship between TSC, treatment response, and survival outcomes, we were able to demonstrate the clinical relevance of this novel parameter in OPSCC. The results of this study may pave the way for studies investigating personalized treatment strategies based on the assessment of the tumor-stroma contact surface, ultimately improving patient care and outcomes in this challenging disease. However, the biological mechanisms underlying the correlation between TSC and response to radiotherapy remain to be elucidated in future studies.

## CRediT authorship contribution statement

**Justus Kaufmann:** Data curation, Formal analysis, Investigation, Software, Visualization, Writing – original draft, Writing – review & editing. **Maximilian Haist:** Investigation, Methodology, Software, Visualization, Writing – original draft. **Ivan-Maximiliano Kur:** Conceptualization, Investigation, Methodology, Writing – original draft. **Stefanie Zimmer:** Data curation, Writing – original draft. **Jan Hagemann:** Writing – original draft. **Christoph Matthias:** Writing – original draft. **Stephan Grabbe:** Writing – original draft. **Heinz Schmidberger:** Writing – original draft. **Andreas Weigert:** Writing – original draft. **Arnulf Mayer:** Conceptualization, Formal analysis, Supervision, Visualization, Writing – original draft, Writing – review & editing.

## Declaration of competing interest

J. Kaufmann, M. Haist, I. Kur, S.Zimmer, J. Hagemann, C. Matthias, S. Grabbe, H. Schmidberger, A. Weigert and A. Mayer declare that they have no competing interests.

## Appendix Supplementary materials

Image, application 1

Image, image 2
