# Supplementary material for: Tumor-stroma contact ratio - a novel predictive factor for tumor response to chemoradiotherapy in locally advanced oropharyngeal cancer
Source: Transl Oncol. 2024 Jun 3;46:102019. doi: 10.1016/j.tranon.2024.102019 (PMC11190748; doi:10.1016/j.tranon.2024.102019)
Supplement: Supplementary file 1 [file mmc1.docx]

**Supplementary Material**

**Supplementary Table 1: Key Resources and Materials**

| **Reagent or Resource** | **Manufacturer** | **Identifier** |
| --- | --- | --- |
| **Chemicals and Reagents** | | |
| Xylol (Isomere) | Carl-Roth GmbH +Co. KG (in the following termed Carl-Roth GmbH) | 9723.5 |
| 99,8 % EtOH | Carl Roth GmbH | K928.3 |
| 96 % EtOH | Carl Roth GmbH | T171.4 |
| 70 % EtOH (dilution from 99,8 % ETOH) | Carl Roth GmbH | - |
| Tris-HCl (Pufferan) p.a. | Carl Roth GmbH | 9090.3 |
| NaCl (99,8%) | Carl Roth GmbH | 9265.1 |
| Formalin solution (neutral buffered 10 %) | Sigma-Aldrich | HT501128-4L |
| Fluorescence Mounting Medium | Agilent DAKO | S302380-2 |
| OPAL Polaris 7-Color (manual Kit) | Akoya Biosciences | NEL861001KT |
| Opal Polymer HRP (Ms+Rb) | Akoya Biosciences | ARH1001EA |
| Opal 480 | Akoya Biosciences | - |
| Opal 520 | Akoya Biosciences | - |
| Opal 570 | Akoya Biosciences | - |
| Opal 690 | Akoya Biosciences | - |
| Opal 620 | Akoya Biosciences | - |
| Opal 780 | Akoya Biosciences | - |
| TSA-Dig | Akoya Biosciences | - |
| DAPI | Akoya Biosciences | FP1490 |
| Antibody Diluent | Akoya Biosciences | ARD1001EA |
| Amplification Diluent | Akoya Biosciences | IF1498 |
| AR9 Buffer | Akoya Biosciences | AR900250ML |
| ImmEdge_Pen (hydrophobic barrier pen) | Vector Laboratories | H-4000 |
| Aqua_rinsing solution (Ampuwa) | Fresenius Kabi Deutschland | 1088811 |
| AVS Titrinorm pH 4 | Avantor delivered by VWR | 32095.264 |
| AVS Titrinorm pH 7 | Avantor delivered by VWR | 32096.267 |
| AVS Titrinorm pH 9 | Avantor delivered by VWR | 32039.261 |
| EDTA >99 % | Carl Roth GmbH | CN06.2 |
| Tris Pufferan >99 % p.a. | Carl Roth GmbH | 4855.2 |
| Eukitt (mounting medium) | Avantor delivered by VWR | 03989-100 ml |
| PBS (without Ca^2+^, Mg^2+^) | Bio&Sell | BS.L.182-50 |
| tri-Natriumcitrate (Dihydrat) | Carl Roth GmbH | 3580.1 |
| Hydrogen peroxide 3 % | neoLab | LC-10300.1 |
| Citric acide (Monohydrate) | Carl Roth GmbH | 5110.1 |
| Hematoxylin | Agilent (DAKO) | CS700 |
| ImmPRESS HRP Horse Anti-Mouse IgG (Kit) | Vector Laboratories | MP-7402 |
| ImmPRESS HRP Horse Anti-Rabbit IgG (Kit) | Vector Laboratories | MP-7401 |
| ImmPACT_DAB Substrate, HRP | Vector Laboratories | SK-4105 |
| Dulbecco`s PBS w/o Ca,Mg | Sigma-Aldrich | D8537-500 ml |
|  |  |  |
| **Antibodies and Proteins** | | |
| Pan Cytokeratin Plus [AE1/AE3+5D3] | Biocare Medical | CM162A |
| p16 INKa [E6H4] | CINtec (Roche) | 659444100 |
| Ki67 [SP6] | abcam | ab16667 |
| CD8a [D8A8Y] | Cell Signaling | 85336S |
| CD271 [SA39-02] | Thermo Fisher Scientific | MA5-31968 |
| PD-L1 [E1L3N] | Cell Signaling | 13684T |
| **Critical Commercial Instruments, Consumables, Kits and Assays** | | |
| Vectra Polaris Imaging System | Akoya Biosciences | CLS143455 |
| Nanozoomer 2.0HT | Hamamatsu Photonics | C9600 |
| Thermomixer Compact | Eppendorf | 535025873 |
| pH-meter (Lab 850) | Schott Instruments | 6110160 |
| Balance (Adventurer Pro AV2101) | Ohaus | 8727143362 |
| Fine balance (Extend) | Sartorius | ED124S-OCE |
| Stirrer (MR3000) | Heidolph | 119907925 |
| Microwave oven | Bosch | HMT75M451 |
| Pipet_(Eppendorf research 1000 µl) | Eppendorf | 4855156 |
| Pipet_(Eppendorf research 100 µl | Eppendorf | 1264836 |
| Pipet_(Eppendorf research 10 µl | Eppendorf | 4117065 |
| Stain Tray black | Carl-Roth GmbH | HA51.1 |
| Timer | Macherey-Nagel | 140277-006 |
| Heating oven (TV-40u) | Memmert (Schwabach) | 770633 |
| Freezer | Liebherr | 7083245-00 |
| Shaker (Polymax 1040) | Heidolph | 543-42205-00 |
| Färbekästen aus Kalk  Soda Glas | Carl-Roth GmbH | H554.1 |
| Dye box for 10 slides | Carl-Roth GmbH | H552.1 |
| Dye box and inserts (PMP) | Carl-Roth GmbH | 2290.2 / 2291.2 |
| Pipet tips (10/20 µl XL graduated tip) | Starlab Group | S1110-3700 |
| Pipet tips (1000 µl blue graduated tip | Starlab Group | S1111-6701 |
| Pipet tips (200 µl yellow tip) | Starlab Group | S1111-0706 |
| Lab gloves Nitra Touch | Ansell | 4400053 |
| U-gloves Nitril Blue | Abena Nova | 290419 |
| Cover glass tweezer 18/8 | Carl-Roth GmbH | K718.1 |
| Powder funnel | Vitlab | 71094 |
| Rotilabo  Weighing bowls (89*89mm) | Carl-Roth GmbH | 2150.1 |
| Alufoil | Carl-Roth GmbH | AAI 76.1 |
| Lab bottle (1000 ml) | Schott Duran | 21820545 |
| Stirring rods | Carl-Roth GmbH | PK77.1 |
| Kimtech science | Kimberly-Clark | 7552 |
| 3M KCl | SI Analytics | - |
| 4 N NaOH | Carl-Roth GmbH | T198.1 |
| 1 M HCl | Carl-Roth GmbH | K025.1 |
| Cellstar Tubes (15ml) | Greiner Bio-one | 188271 |
| Reaction tubes 1,5ml | Greiner Bio-one | 618201 |
| Microscope slides ThermoScientific (Superfrost Plus) | Menzel GmbH | 1800AMNZ |
| **Biological Samples** | | |
| Tissue microarray | Institute of Pathology, University Medical Center Mainz | N/A |
| FFPE tissue blocks | Institute of Pathology, University Medical Center Mainz | N/A |
| **Deposited Data** | | |
| Quantitative data table | Uploaded on Dryad:<https://doi.org/10.5061/dryad.95x69p8p8> |  |
| Single-cell coordinates table | Uploaded on Dryad:<https://doi.org/10.5061/dryad.95x69p8p8> |  |
| **Software and Algorithms** | | |
| ImageJ (Fiji version 2.0.0) | <https://imagej.net/> | N/A |
| QuPath version 0.3.2. | <https://qupath.github.io/> | (19) |
| R version 4.0.3 | <https://cran.r-project.org/bin/windows/base/> | (23) |
| R studio desktop, version 1.1.423 | <https://www.rstudio.com/> | (24) |
| Spatial analysis | [www.spatstat.org](http://www.spatstat.org) | (20) |
| Survival R package | <https://cran.r-project.org/web/packages/survival/index.html> | N/A |
| Pheatmap package | <https://cran.r-project.org/web/packages/pheatmap/index.html> | N/A |
| The Human Protein Atlas | <http://www.proteinatlas.org/> | (61) |

**Supplementary Table 2.** Multiplex IF protocol including staining sequence, applied buffers, antibodies and fluorophores as well as resulting staining patterns.

| No | Antigen retrieval buffer | Antigen (clone) | Primary AB  (species, dilution, incubation) | Fluorochrome (Cat.-No.) | Fluorochrome  (dilution, incubation) | Staining pattern |
| --- | --- | --- | --- | --- | --- | --- |
| 1 | AR2 (Tris-EDTA/pH9) | Ki67 [SP6] | rabbit (mono), 1:400, for 1 h at 28 - 30°C | OPAL690 | 1:300, for 10min at 24 - 26°C | Nuclear |
| 2 | AR2 (Tris-EDTA/pH9) | Pan-Cytokeratin  [AE1/AE3 +5D3] | mouse (mono), 1:100, overnight at 4°C | OPAL480 | 1:200, for 10min at 24 - 26°C | Membranous/ cytoplasm |
| 3 | AR2 (Tris-EDTA/pH9) | CD271  [SA39-02] | Rabbit (mono), 1:100, for 1 h at 28 - 30°C | OPAL620 | 1:300, for 10min at 24 - 26°C | Membranous |
| 4 | AR2 (Tris-EDTA/pH9) | PD-L1 [E1L3N] | rabbit (mono), 1:200, overnight at 4°C | OPAL520 | 1:200, for 10min at 24 - 26°C | Membranous |
| 5 | AR2 (Tris-EDTA/pH9) | p16 [E6H4] | mouse  (mono), RTU, for 1 h at 28 - 30°C | OPAL570 | 1:200, for 10min at 24 - 26°C | Nuclear / cytoplasm |
| 6 | AR2 (Tris-EDTA/pH9) | CD8 [D8A8Y] | rabbit (mono), 1:200, overnight at 4°C | TSA-Dig and OPAL780 | 1:100 for 10min at 24 - 26°C and 1:25 for 60min at 24 - 26°C | Membranous |
| 7 | N/A | DAPI | for 5min at 24-26°C | N/A | N/A | Nuclear |
