# Supplementary figures and images for: Tumor-stroma contact ratio - a novel predictive factor for tumor response to chemoradiotherapy in locally advanced oropharyngeal cancer
Source: Transl Oncol. 2024 Jun 3;46:102019. doi: 10.1016/j.tranon.2024.102019 (PMC11190748; doi:10.1016/j.tranon.2024.102019)

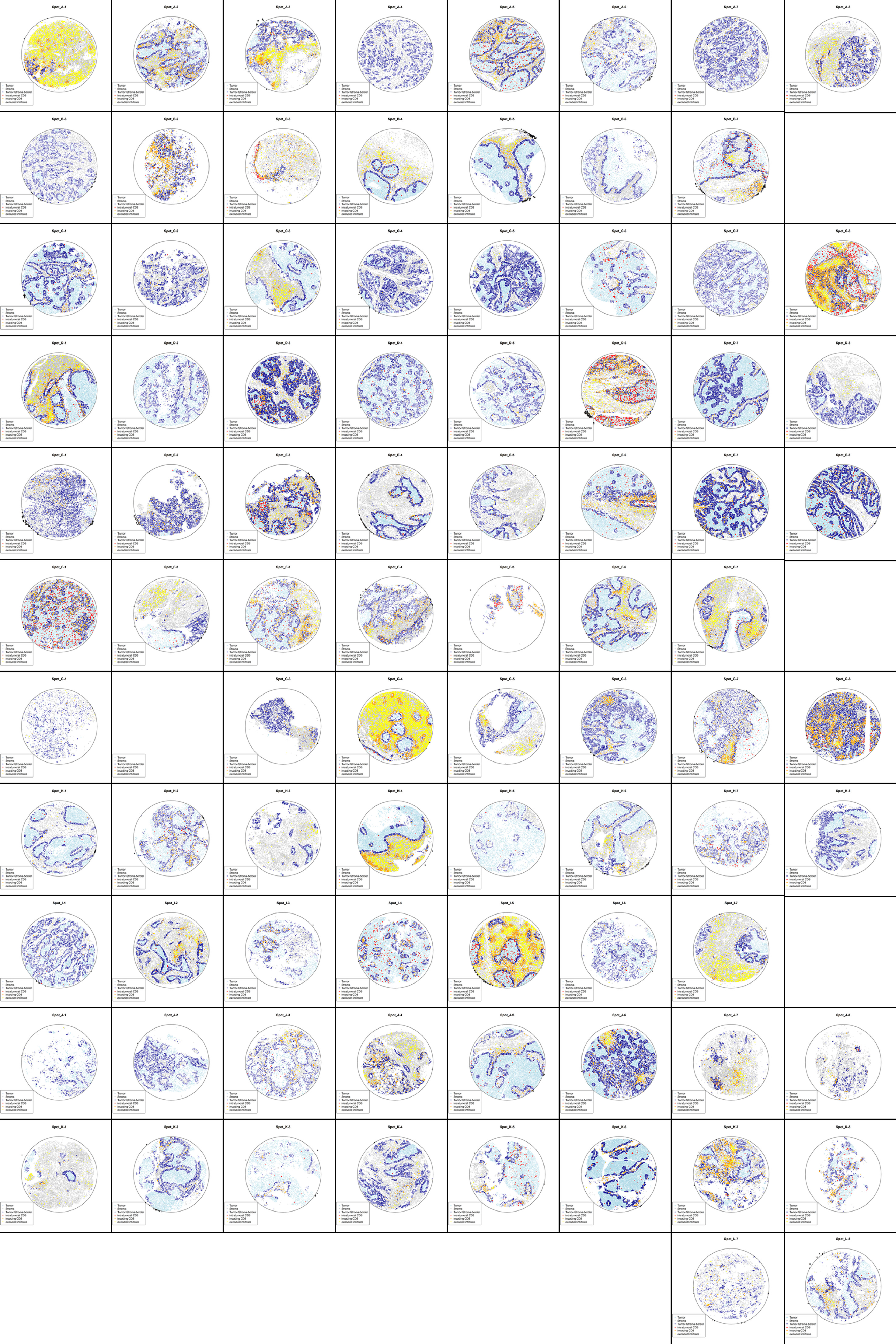

Supplement: Supplementary file 2 [file mmc2.jpg]
